# Brain–Computer Interface Training Enhances Attention Function via Modulating Frontoparietal Connectivity: Evidence From Functional Near‐Infrared Spectroscopy

**DOI:** 10.1155/np/8133428

**Published:** 2026-01-05

**Authors:** Yuhong Huang, Qian Ding, Zhenghong Chen, Jing Chen, Yawen Li, Lu Chen, Shantong Yao, Yue Lan, Guangqing Xu

**Affiliations:** ^1^ Guangdong Cardiovascular Institute, Guangdong Provincial People’s Hospital, Guangdong Academy of Medical Sciences, No. 106 Zhongshan Road II, Guangzhou, 510080, Guangdong, China, gdghospital.org.cn; ^2^ Department of Rehabilitation Medicine, Guangdong Provincial People’s Hospital, Guangdong Academy of Medical Sciences, Southern Medical University, No. 106 Zhongshan Road II, Guangzhou, 510080, China, gdghospital.org.cn; ^3^ Department of Rehabilitation Medicine, The First Affiliated Hospital, Sun Yat-sen University, Guangzhou, China, sysu.edu.cn; ^4^ Department of Rehabilitation Medicine, Guangzhou First People’s Hospital, School of Medicine, South China University of Technology, Guangzhou, China, scut.edu.cn

**Keywords:** attention, brain computer interface, effective connectivity, frontoparietal networks, functional near-infrared spectroscopy

## Abstract

**Objective:**

Attention is a critical cognitive function impaired in various neurological disorders, and brain–computer interface (BCI) training shows potential for cognitive improvement. However, the neural mechanisms of BCI training on attention networks remain unclear. This study investigated the effects of BCI training on attention and the underlying neural mechanisms in healthy young adults.

**Methods:**

Thirty healthy young adults participated in this study. Attention function was assessed using the attention network test (ANT), while brain activation and connectivity were measured using functional near‐infrared spectroscopy (fNIRS). Participants underwent the ANT and fNIRS assessments before and after BCI training.

**Results:**

BCI training significantly improved the efficiency of the executive control network (*p* = 0.016). Nodal efficiency in the right posterior parietal cortex (PPC) was decreased (*p*  = 0.044). In the resting state, effective connectivity (EC) analysis showed decreased connectivity from the right PPC to the left PPC in the resting state (*p*  = 0.047). In the task state, the EC from the right prefrontal cortex (PFC) to the right PPC was significantly increased (*p*  = 0.016), and the connectivity from the left PFC to the right PFC was significantly decreased (*p*  = 0.023).

**Conclusion:**

BCI training optimized connectivity within frontoparietal networks (FPNs), leading to enhanced executive control function. These findings suggest that BCI training could be an effective cognitive intervention for improving the function of FPNs. Future studies should explore the long‐term effects of BCI training and its potential application in clinical populations, such as patients with attention deficit hyperactivity disorder and stroke.

## 1. Introduction

Many cognitive processes involve the participation of attention, which plays an indispensable role in filtering out information that is irrelevant to the current tasks and enhancing the ability to focus on important information [1, 2]. Indeed, an integrated function of attention could be divided into three subfunctions: alerting, orienting, and executive control [3]. The attention network test (ANT), designed by Fan et al. [4], incorporates the orienting task, flanker task, and conflict task, which measure the function of alerting, orienting, and executive control networks simultaneously. Numerous studies demonstrated its effectiveness in identifying deficits in specific attentional components in different diseases[5–7].

Impairments in attention are commonly observed in a variety of neurological disorders, including attention‐deficit hyperactivity disorder (ADHD) [8], stroke [9], and melancholia [10]. Furthermore, the recovery process is closely associated with their attentional functions. Cognitive and motor function recovery often complement each other [11]. Studies have shown that attention can participate in and influence the recovery of motor function in stroke patients [12]. In healthy adults, attention is essential for various aspects of daily life and learning. Attentional processes are implicated in learning across multiple domains, and executive functions serve as high‐order cognitive processes that facilitate adaptation to the external environment and also underpin word reading and reading fluency [13, 14]. Consequently, impairments in executive functions and inattention may represent key contributors to reduced learning efficiency among young individuals. These findings underscore the importance of attention training for both healthy individuals and patients.

A functional magnetic resonance imaging study by Fan et al. revealed activation of the frontoparietal network (FPNs), including regions such as the dorsolateral prefrontal cortex (DLPFC), posterior parietal cortex (PPC), and frontal eye fields (FEF), during the ANT task [3]. This finding highlights the critical role of the FPNs in attention function [15]. Additionally, the studies that decreased FPNs’ functional connectivity in people with inattentiveness [15], as well as the abnormal activities of cingulate‐gyral‐frontal‐parietal in patients with ADHD [16], further support the essential involvement of the FPNs in attention. It can be seen that the integrity of the structure and function of the FPNs is a necessary condition for maintaining normal attentional performance. Therefore, enhancing FPNs’ function may represent a potentially effective strategy for improving attention function. Previous studies have shown that physical exercise and serious gaming activities can improve attention and decision‐making abilities in young individuals, which is associated with the enhanced FPNs function and emphasized that the focus on motor control may serve as an effective stimulus for strengthening FPNs connectivity [17]. However, the underlying neural circuitry mechanisms involved in enhancing FPNs function require further investigation.

The neurofeedback mechanism in brain–computer interface (BCI) training is in line with the focus on motor control mentioned above. Specifically, brain activity is detected by BCI and subsequently translated into external commands that are transmitted to external movement devices. External devices can provide state‐dependent sensory feedback to BCI users, which is referred to as a closed‐loop BCI system [18, 19]. BCI training, a widely applied modality in neurorehabilitation, typically combines BCI with motor imagery training to form a comprehensive rehabilitation program [20]. Recent studies indicated that BCI not only possesses the potential for the motor rehabilitation in patients with movement disorders but also could be utilized to improve attention in stroke patients, which may be attributed to neuroplasticity induced by BCI [20, 21]. However, brain function was absent in these studies. Consequently, the neural mechanisms by which BCI training improves the functions of attention networks remain unclear.

Participants typically engage multiple complex cognitive processes during BCI training, which involve the activation of several cortical regions, such as the bilateral prefrontal cortices and parietal cortices (components of the FPNs). And the enhanced connectivity in attention‐related pathways induced by BCI could be observed in many studies [22, 23]. Therefore, we used the brain functional monitoring device to examine the functional changes in the FPNs before and after BCI training. Functional near‐infrared spectroscopy (fNIRS) serves as a noninvasive technique extensively utilized to measure brain activity and functional connectivity across various cortical areas at the same time [18]. Granger causality analysis can provide directional functional interactions (i.e., effective connectivity, EC) between brain regions [24]. Therefore, we employed Granger causality analysis to explore the information flow of FPNs during attention tasks.

In this study, we explored how BCI training impacts attention network functions in healthy young adults. We proposed the following hypotheses: (1) BCI training will boost the attention network efficiency; (2) BCI training will enhance brain activation of some cortices in FPNs during the ANT; and (3) BCI training will strengthen EC and functional connectivity within the FPNs during the ANT.

## 2. Methods

### 2.1. Participants

The study included 30 healthy young adults, comprising 14 males and 16 females, with a mean age of 22.0 years (SD = 1.48). All participants were right‐handed, as determined by the Edinburgh Handedness Inventory. And they had no history of neurologic diseases or injuries to the head or hands. Written informed consent was obtained from each participant before the experiment. The Guangdong Provincial People’s Hospital Human Research Ethics Committee has approved this research (KY2023‐1079‐02) and we finished the process of registration at the Chinese Clinical Trial Registry (ChiCTR2500097678). The research was conducted in accordance with the Declaration of Helsinki.

### 2.2. Experimental Procedure

**Figure 1:**
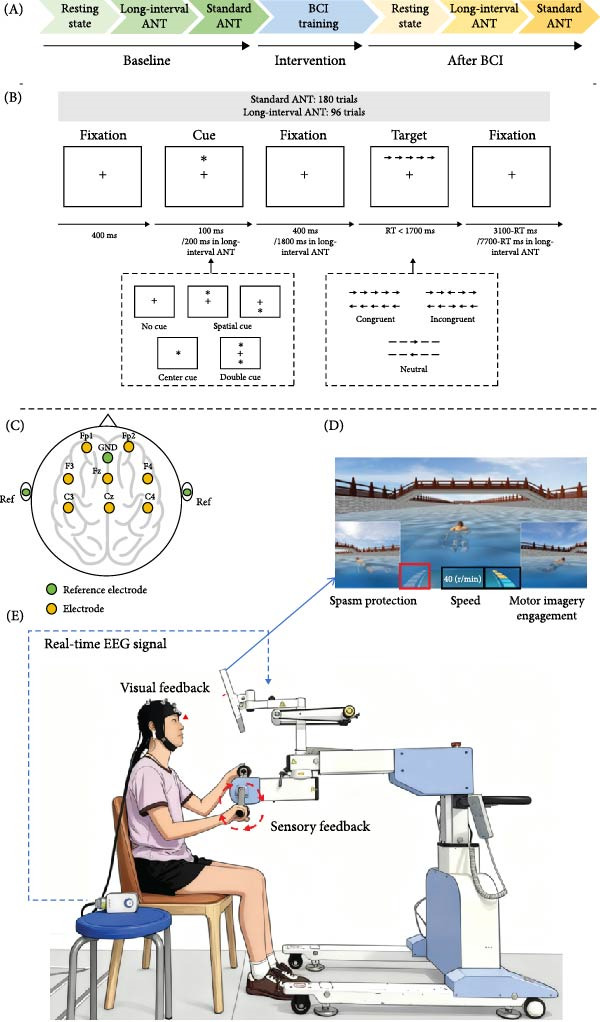
Experimental procedure. (A) Experimental procedure. The baseline assessment was conducted on the first day, and BCI training was continued on the second day. BCI training session lasted for 10 min. The behavioral data of ANT and functional near‐infrared brain imaging were collected before and after the BCI training. (B) Schematic of the attention network test. At the beginning of each trial, a fixation point lasting 400 ms was presented in the center of the screen. This was followed by a cue stimulus that lasted 100 ms in the standard ANT (200 ms in the long‐interval ANT). Then, the fixation reappeared (lasting 400 ms in standard ANT and 1800 ms in long‐interval ANT). After that, the target stimulus was presented, and the subject had a maximum RT of 1700 ms. Fixation points appeared immediately after the response, and their duration correlated with the length of the RT. (C) The location of EEG electrodes. (D) Illustration of the motor imagery animation. In the animation, the swimming speed of the character varies with the participant’s level of motor imagery engagement. The left block‐shaped progress bar represents spasm protection, while the right block‐shaped progress bar indicates the participant’s current level of motor imagery engagement. (E) Illustration of the closed‐loop system.

The baseline assessment was conducted before the BCI training. Each participant received BCI training for 10 min. The ANT and fNIRS data were collected again after the BCI training (Figure 1A).

#### 2.2.1. ANT

E prime 3.0 experiment software were used for programing and presenting the ANT. Participants sat in a quiet room, with their eyes 65 cm away from the screen of laptop. A total of 291 trials were completed, consisting of three phases: 15 practice trials, 96 long‐interval ANT trials (a modified version of ANT with extended intertrial intervals to allow blood oxygen levels to return to baseline), and 180 standard ANT trials. A complete trial consists of five steps including fixation, cue stimulus and target stimulus [4]. First, a fixation was displayed for 400 ms. Then one of the cue stimuli appeared for 100 ms. After the cue signal, there was a short fixation period of 400 ms. The target stimuli appeared 1700 ms, and participants need to press the keyboard “F” to the left or “J” to the right according to the direction of the target arrow. The arrow disappeared immediately after the participants responded, and a post‐target fixation was present for a variable duration, calculated as 3500 ms minus the duration of the first fixation minus the reaction time (RT). It is worth mentioning that the duration of the cue stimuli and the second fixation in the long‐interval ANT were 200 and 1800 ms, respectively. The duration of the postresponse fixation was changed to 8100 ms minus the duration of the first fixation minus RT. Each trial lasted for 4000 ms in standard ANT or 10,100 ms in long‐interval ANT. The accuracy and RT were recorded. For details, see Figure 1B.

For each participant, the average RTs were computed across the 12 possible combinations of the different cue types (double cue, no cue, spatial cue, central cue) and the different flanker types (neutral, congruent, incongruent). The efficiency of attention networks was defined as the differences of RTs above [4]. The specific calculation formula is as follows:

Efficiencyalerting=RTno cue− RTdouble cue ,

Efficiencyorienting=RTcentral cue − RTspatial cue ,

Efficiencyexecutive=RTincongruent− RTcongruent.

Only correct trials were included in the calculations, with RTs ranging from more than 150 ms to less than 1000 ms.

#### 2.2.2. BCI Training

The L‐B300 EEG acquisition rehabilitation training device (Xiangtan Mailian Medical Equipment Co., Inc., Hunan, China) consists of an EEG acquisition system, a motor training host, and software for evaluating training data. The placement of the conductive electrodes followed the international 10–20 system (Figure 1C). Prior to the training, participants were informed that the motor imagery task is swimming. After the EEG cap was positioned and all electrode impedances were reduced to the acceptable range, the BCI training equipment was initiated. Participants grasped the handles and awaited the start signal from the device to begin motor imagery. A yellow progress bar on the right side of the screen indicated the real‐time engagement level of the participant’s motor imagery (Figure 1D). Once the threshold was reached, the handle began to rotate, gradually increasing to a maximum speed of 40 r/min. During this process, the handle provided sensory feedback to the participant, while the animation of a character swimming accelerated, offering visual feedback (Figure 1E). As the swimming distance increased, the scenery around the character in the animation also changed. This multimodal feedback encouraged participants to continuously adjust their motor imagery strategies to optimize BCI performance.

The BCI training intensity was divided into 15 levels, with higher levels requiring stronger event‐related desynchronization to achieve the threshold. For our experiment, the researchers set the training intensity to level 10. This setting ensured that the BCI feedback was mainly determined by the participant’s degree of engagement in motor imagery. All the participants received 1 min of practice and 10 min of training.

### 2.3. fNIRS

#### 2.3.1. Data Acquisition

In this study, we utilized a 63‐channel fNIRS device (NirScan, Danyang Huichuang Medical Equipment Co., Inc., Jiangsu, China), comprising 24 emitters and 24 receivers, to monitor changes in cerebral oxy‐Hb and deoxy‐Hb concentrations during ANT tasks and resting state. The device employed three wavelengths of near‐infrared light (730, 808, and 850 nm). The channel distribution is depicted in Figure 2.

Figure 2Distribution of fNIRS channels. (A) Regions of interest. The green circles represent the source, and the yellow circles represent the detectors. The lines between the green and yellow circles indicate the channels, and the number represents the channel number. The fNIRS channels are distributed in the bilateral parietal and bilateral prefrontal cortices. (B) fNIRS 63‐channel montage placement.

**Figure:**
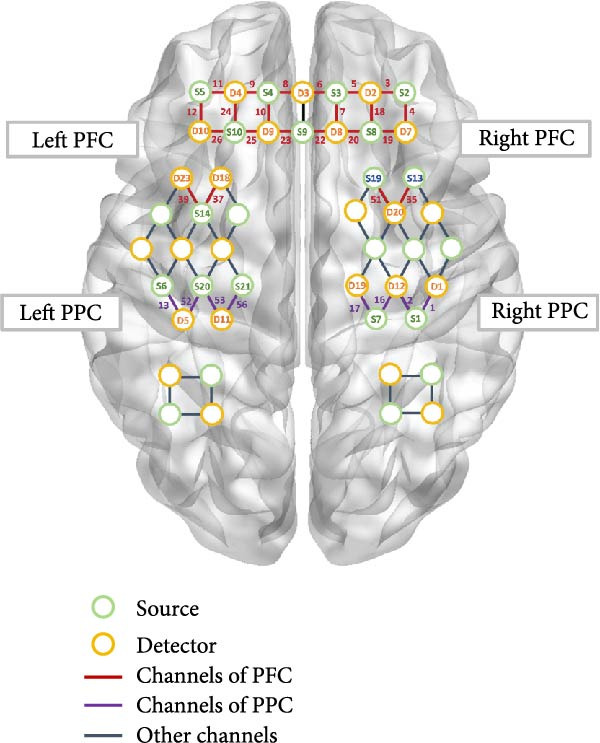
(A)

**Figure:**
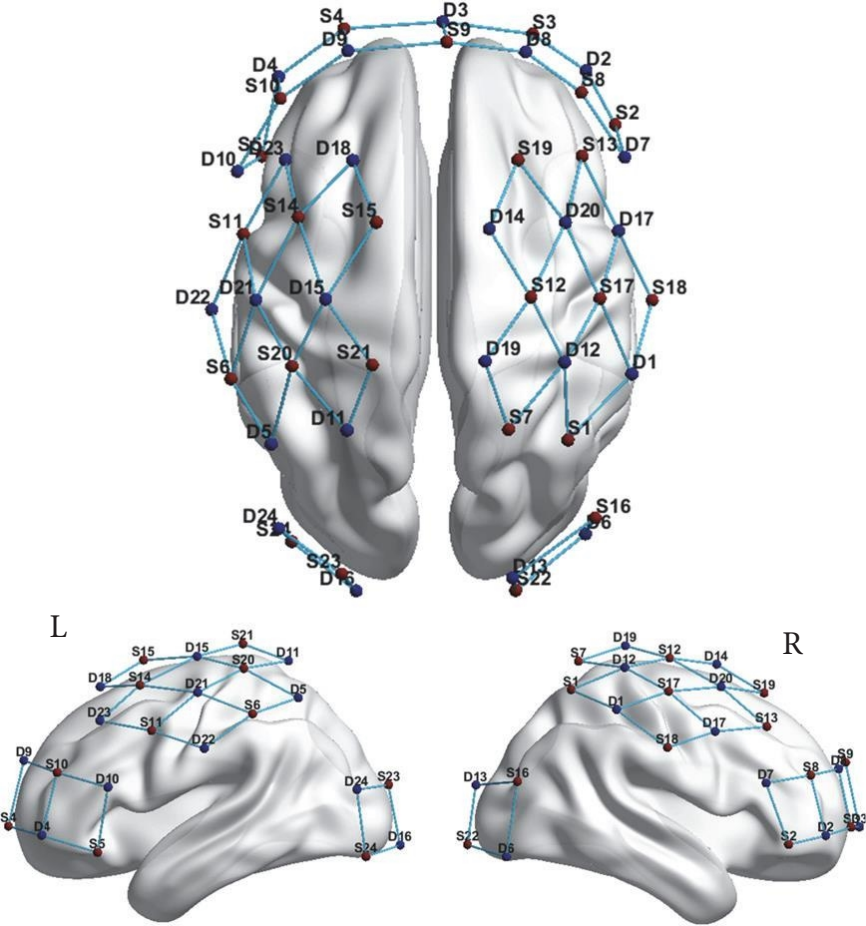
(B)

Regions of interest (ROIs) were selected via Polhemus PATRIOT digitizer channel registration analyses. Before the tasks began, the participants were asked to keep the fNIRS cap in place as experimenters carefully removed the optodes. The center point of the head (i.e., the Cz point) was determined using the measuring tape by measuring the distance between the left and right earlobes and from the nasion (the midpoint between the eyes) to the inion (the protrusion at the back of the skull). After identifying the Cz point, a magnet was placed at this location. Then the participant was positioned to maintain a 10 cm distance between the inion and the transmitter. Following this, the nasion, left tragus and right tragus, inion, and Cz were measured with a stylus. Subsequently, the remaining optical fiber points were measured sequentially. The ROIs selected were the right PFC (channels 3–7, 18–20, 22, 35, 51), left PFC (channels 8–12, 23–26, 37, 39), right PPC (channels 1, 2, 16, 17), and left PPC (channels 13, 52, 53, 56). Channels with over 50% overlap in each ROI were averaged using MRIcro registration analysis [25, 26].

The sampling frequency was 11 Hz. A chin bandage was utilized to secure the position of the fNIRS cap, thereby minimizing cap movement and ensuring intimate contact between the light source and detector probes and the scalp to ensure optimal signal acquisition. This method was employed to enhance the efficiency of light‐to‐tissue coupling. To ensure that the signal gain was within an appropriate range, gain quality checks were conducted prior to data acquisition, thereby avoiding both under‐gain and over‐gain in the recorded data.

#### 2.3.2. Data Analysis

The concentration fluctuations of delta‐oxygenated HbO_2_ were calculated based on changes in detected light intensity, using the modified Beer–Lambert Law and assuming constant scattering [27]. Differential pathlength factors (DPFs) were calculated according to Scholkmann et al. [28, 29].

DPFλ,A=223.30.056240.001245+A0.8493+−5.723×10−7λ3+λ2+−0.9025λ,

where *λ* represents the wavelengths, and A represents ages [29].

The DPF values used in the preprocessing pipeline are 6.0776 (730 nm), 5.7758 (808 nm), and 5.0003 (850 nm). To mitigate the impact of large‐scale artifacts, such as motion artifacts and physiological noises, principal component analysis (PCA) was applied to the optical density (OD) data. The components that collectively accounted for the top 97% of the total variance were removed. The corrected OD data were subsequently reconstructed from the remaining principal components [30]. The physiological signals were removed using the low‐pass filter (0.1 Hz). The low‐frequency drift was removed by a high‐pass filter (0.01 Hz) [31]. The changes in blood oxygen concentration in the ROI channels were calculated for statistical analysis.

We used the data recorded in long‐interval ANT to calculate brain activation. The data were extracted from a period of 1 s prior to and 9.1 s following the onset of the cue on each trial. The relative change in activation was calculated by subtracting the value between the two markers [3].

We calculated brain activation according to Fan et al. by subtracting between conditions [3]. The trails used for brain activation analysis met all of the following criteria: the response was correct, and the RT was longer than 150 ms and less than 1000 ms. The formulas were as follows:

Activationalerting=HbOdouble cue − HbOno cue,

Activationorienting=HbOspatial cue −HbOcentral cue,

Activationexecutive= HbOincongruent− HbOcongruent.

Functional connectivity between the cortices in the FPNs was assessed by calculating coherence using MATLAB scripts during resting‐state and standard ANT conditions. The squared coherence was calculated using a Welch‐averaged, modified periodogram method [32]. Connectivity matrices were subsequently subjected to Fisher’s z‐transformation to normalize the data to a Gaussian distribution [31].

Graph theory analysis was performed using the GRaph thEoreTical Network Analysis (GRETNA) toolbox. Weighted, undirected networks were constructed based on coherence values. To comprehensively characterize the brain network, we integrated metrics across the full threshold range (from 0.1– to 0.4, with an interval of 0.05) to obtain the area under the curve characterizing the brain network. Based on graph theory, we calculated the average efficiency of connections between all pairs of channels within each ROI. This measure, termed local network efficiency, indicates how efficiently information is transmitted within each ROI [33]. Nodal efficiency and degree centrality are commonly used metrics to calculate local network efficiency [34].

Nodal efficiency is calculated as the inverse of the harmonic mean of the shortest path lengths from a given node to all other nodes within the brain network. This measure indicates the capacity of a node to efficiently disseminate and receive information across the entire brain [32]. It can be calculated by the following formula:

Ei=1N−1∑j∈N, j≠i1Di,j,

where *D* (*i*, *j*) is the shortest path length between node *i* and node *j*, and *N* is the number of nodes in the network.

Degree centrality is a graph metric that assesses the importance of each node in a brain network, evaluating the connectivity strength to every voxel. It distinguishes the node with the most connections by calculating the number of direct links to others to determine the relative importance of nodes within the network [35]. The degree of centrality was calculated using the following formula:

DCi= ∑j=1Naij.

The element *a*_*ij*_ of the adjacency matrix represents the connection or edge from node *i* to node *j*, which is 0 if no edge exists and is nonzero for an edge with a weight *a*_*ij*_.

The HERMES toolbox was used for the Granger causality analysis. First, fNIRS data are preprocessed, including noise removal and motion artifact correction, to ensure data quality. Assume that *X*_*A*_ and *X*_*B*_ represent the time series of ROI_*A*_ and ROI_*B*_, respectively. Linear autoregressive models are constructed based on their past observations to estimate prediction errors. The specific models are as follows:

XAt= a0+∑i=1paiXAt−i+εAt,

XBt= b0+∑i=1pbiXBt−i+εBt.

Here, *p* is the model order determined by the Bayesian information criterion (BIC), and *ε*_*A*_(*t*) and *ε*_*B*_(*t*) are white noise processes. The autoregressive model of *X*_*A*_ is augmented by including past observations of *X*_*B*_; similarly, the autoregressive model of *X*_*B*_ is augmented by including past observations of *X*_*A*_. By comparing the prediction errors of the augmented models with those of the original models, it is tested whether the past observations of one channel significantly reduce the prediction error of the other channel, thereby defining the causal relationship between the two channels. The specific formulas are as follows [24]:

GB→A=  lnCOVεBACOVεA,

GA→B=  lnCOVεABCOVεB.

Here, COV(*ε*_*BA*_) and COV(*ε*_*AB*_) represent the residual variances of the augmented models.

### 2.4. Statistical Analysis

IBM SPSS Statistics 27 was employed for statistical analysis. The Shapiro–Wilk test was conducted to assess the normality of the data, which was confirmed to be normally distributed.

To assess ANT performance, paired *t*‐tests were conducted to evaluate changes in the efficiency of three attention networks (alerting, orienting, and executive control) before and after BCI training.

A repeated‐measure [Day (2) × ROI (4)] ANOVA was applied to compare brain activation before and after BCI training in four brain regions. A repeated‐measure [Day (2) × ROI (6)] ANOVA was applied to assess the coherence between the four ROIs within FPNs before and after BCI training. Repeated‐measure [Day (2) × ROI (4)] ANOVAs were applied to assess changes in nodal efficiency and degree centrality before and after BCI training. A repeated‐measure [Day (2) × EC (12)] ANOVA was applied to assess the EC of 12 types of Granger causality before and after BCI training. For all repeated‐measures ANOVAs, the sphericity assumption was verified using Mauchly’s test. When the assumption was violated (*p*  < 0.05), the Greenhouse–Geisser correction was applied, and the adjusted degrees of freedom and *p*‐values are reported. In all ANOVA models above, the Bonferroni correction was applied to adjust for multiple comparisons. The number of comparisons in the ANOVA models was 4 for brain activation, nodal efficiency, and degree centrality; 6 for functional connectivity; and 12 for EC.

The presented *p* values in the results section are all adjusted, with *p* < 0.05 indicating statistical significance.

## 3. Results

### 3.1. Attention Network Test

The paired *t*‐test indicated a significant effect on the executive control network following BCI training (t = −2.557, *p* = 0.016). No significant differences were observed in the orienting network (*p* = 0.586) and the alerting network (*p* = 0.280). The efficiency of the executive control network was enhanced, as shown in Figure 3.

Figure 3Efficiency changes in attention networks after BCI training. (A) After BCI training, the efficiency of alerting network was not significantly changed. (B) After BCI training, the efficiency of orienting network was not significantly changed. (C) After BCI training, the efficiency of executive network was improved significantly.  ^∗^ *p*  < 0.05.

**Figure:**
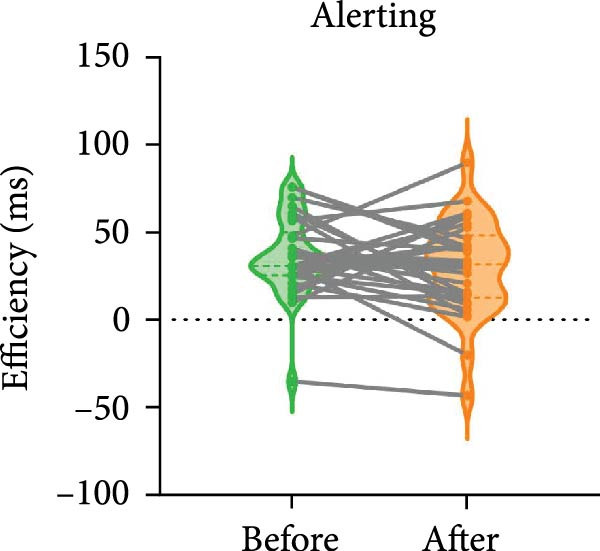
(A)

**Figure:**
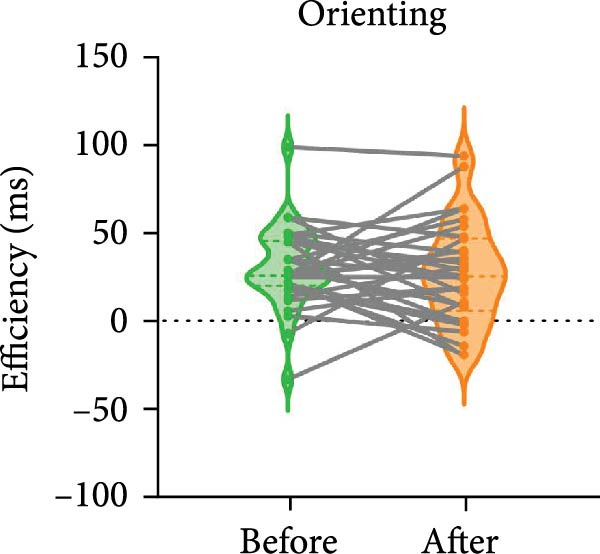
(B)

**Figure:**
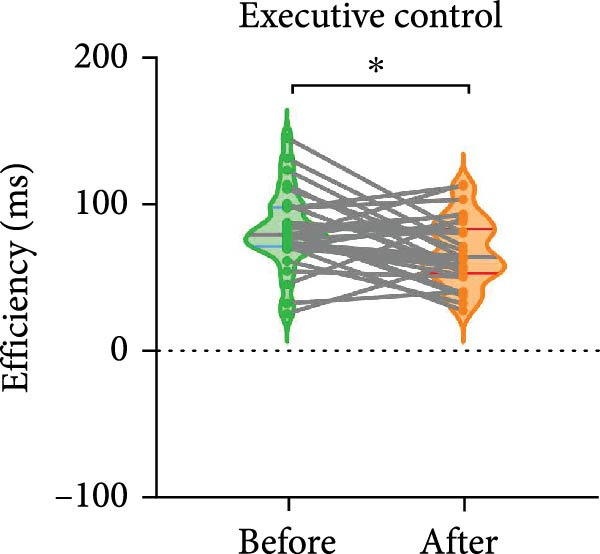
(C)

### 3.2. fNIRS Data

#### 3.2.1. Brain Activation

**Figure 4:**
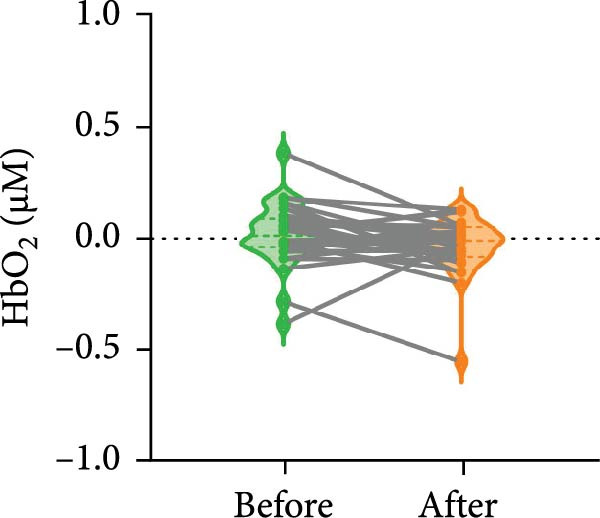
Changes of the HbO_2_ concentration in the right PPC during long‐interval ANT. A decreasing trend in HbO_2_ concentration was observed in the right PPC after BCI training (*p* = 0.067).

The ANOVA model showed no significant main effect of Day [*F*_(1,29)_ = 1.033, *p* = 0.318] and ROI [*F*_(3,27)_ = 0.223, *p* = 0.880], and no significant effect of Day × ROI interaction [*F*_(3,27)_ = 2.815, *p* = 0.056] in the alerting network. Post hoc analysis indicated a decreasing trend of the concentration of HbO_2_ in the right PPC (*p* = 0.067) (Figure 4). The ANOVA model showed no significant differences within the orienting and executive control networks before and after BCI training. [*F*_(1,29)_ = 0.005, *p* = 0.945; *F*_(1,29)_ = 0.036, *p* = 0.850, respectively].

#### 3.2.2. Resting‐State Analysis

**Figure 5:**
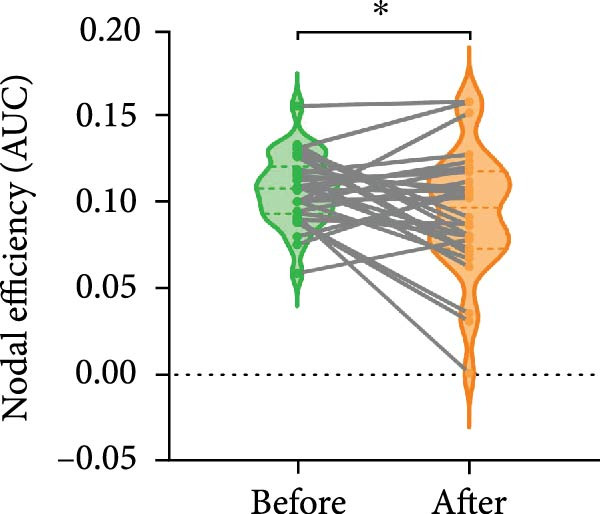
Changes of network efficiency of right PPC in resting after BCI training. After BCI training, the nodal efficiency of right PPC in resting state was decreased significantly.

No significant changes were observed in resting‐state functional connectivity after BCI training [*F*_(1,29)_ = 0.647, *p* = 0.428]. For nodal efficiency in resting‐state, no significant differences were observed in the main effect of Day [*F*_(1,29)_ = 0.854, *p* = 0.363] and ROI [*F*_(3,27)_ = 2.537, *p* = 0.078], and the effect of Day × ROI interaction [*F*_(3,27)_ = 2.706, *p* = 0.065]. Post hoc analysis revealed a significant decrease in nodal efficiency within the right PPC (*p* = 0.044) (Figure 5). For degree centrality in resting‐state, no significant changes were observed [*F*_(1,29)_ = 1.898, *p* = 0.179] after BCI training. For resting‐state EC, the ANOVA model revealed no significant main effect of Day [*F*_(1,29)_ = 0.681, *p* = 0.416] and EC [*F*_(11,19)_ = 1.989, *p* = 0.091], and no significant effect of Day × EC interaction [*F*_(11,19)_ = 1.347, *p* = 0.274]. Post hoc analysis showed that the EC from right PPC to left PPC was significantly decreased (*p* = 0.047) (Figure 6). The EC from right PPC to left PFC was in an increasing trend (*p* = 0.069).

Figure 6Changes of Effective connectivity in resting state after BCI training. (A) Effective connectivity matrices within the bilateral frontoparietal networks before and after BCI training and their differences. (B) Schematic representations of effective connectivity within the frontoparietal network and its differences before and after BCI training.

**Figure:**
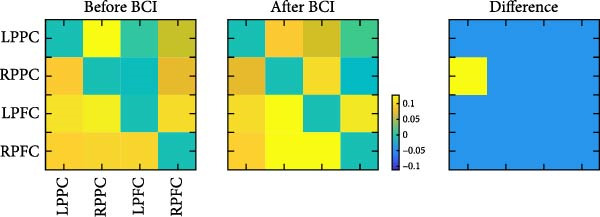
(A)

**Figure:**
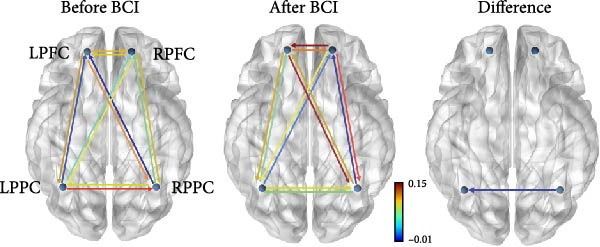
(B)

#### 3.2.3. Task‐State Analysis

No significant changes were observed in task‐based functional connectivity after BCI training [*F*_(1,29)_ = 0.175, *p* = 0.679]. No significant changes were revealed in the nodal efficiency [*F*_(1,29)_ = 0.012, *p* = 0.912] and degree centrality [*F*_(1,29)_ = 0.0001, *p* = 0.990] during ANT after BCI training according to the ANOVA model. For task‐based EC, the significant changes were observed in the main effect of EC [*F*_(11,19)_ = 4.023, *p* = 0.004] and the effect of Day × EC interaction [*F*_(11,19)_ = 3.587, *p* = 0.007]. No significant differences were observed in the main effect of Day [*F*_(1,29)_ = 3.079, *p* = 0.090]. Post hoc analysis showed that the EC from right PFC to right PPC was significantly increased (*p* = 0.016). The EC from left PFC to right PFC was significantly decreased (*p* = 0.023) (Figure 7).

Figure 7Task‐based effective connectivity. (A) Effective connectivity matrices within the bilateral frontoparietal networks before and after BCI training and their differences. (B) Schematic representations of effective connectivity within the frontoparietal network and its differences before and after BCI training.

**Figure:**
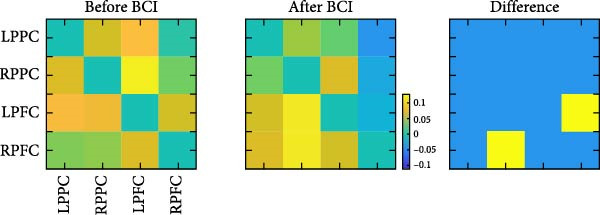
(A)

**Figure:**
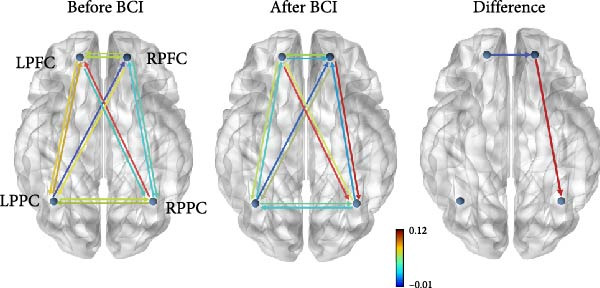
(B)

## 4. Discussion

In this study, we investigated the effects of BCI training on the FPNs functions in healthy young adults. The ANT and fNIRS were employed simultaneously to detect the changes in attention network efficiency and explore the underlying neural mechanism that induced the changes above. The main findings of this study were as follows: (1) the efficiency of the executive control network increased significantly. (2) The brain activation of the alerting network decreased. (3) The EC within the FPNs changed significantly. (4) The network efficiency of the right PPC was decreased significantly.

### 4.1. The Efficiency of the Executive Control Network was Enhanced Significantly After BCI Training

There was a significant enhancement in the efficiency of the executive control network after BCI training. Our findings were in accordance with the previous studies that BCI training had a positive impact on attention [21]. This effect may be attributed to BCI‐based neurofeedback training, which enhances cognitive performance and allows subjects to promptly adapt their attention strategies [36, 37]. The participants drove the BCI device by motor imagery, and the movement of the BCI and the swimming speed of the person on the screen gave feedback to the participants for better BCI motor control. Focusing on motor control during training might be a way to improve specific aspects of executive control in young adults by adjusting the balance between speed and accuracy [17]. It can be seen that BCI training can be used as a potentially effective method to enhance attention and executive ability. In addition to the behavioral functions of the alerting, orienting, and executive control networks, which interact with each other, they also operate relatively independently. Although previous studies [21] have demonstrated that BCI training can enhance the orienting function in stroke patients, our results indicate that the efficiency of the alerting and orienting networks did not change after BCI training in healthy young adults. We speculate that the discrepancy in findings may be attributed to differences in the participants. The structure and function of the FPN in healthy adults are relatively normal [38]. A single session of BCI training may not be sufficient to induce significant improvements in FPNs functions. Additionally, our BCI training device does not involve the execution of spatial orienting tasks, which may also be one of the reasons for the lack of significant enhancement in the orienting network function.

### 4.2. The Activation of Right PPC was in a Decreasing Trend in the Alerting Network

The HbO_2_ concentration of the right PPC in the alerting network was in a decreasing trend after BCI training, which indicates the lower level of brain activation and the higher efficiency of FPNs. The observed decline in blood oxygen levels deviated from our initial hypotheses. However, it is well established that increased brain activation does not invariably correlate with enhanced functional efficiency. This discrepancy may be attributable to several underlying mechanisms, including the compensatory mechanism within the brain, inefficient utilization of neural resources, or functional reorganization of brain activation patterns [39]. The bilateral posterior parietal cortices are involved in spatial attention tasks [40], with the right PPC being one of the key brain regions for spatial attention [41]. Lesions in the right PPC are often associated with symptoms of unilateral spatial neglect [42]. Our results indicate that the efficiency of neural resource utilization within FPNs has increased, characterized by a reduction in HbO_2_ levels and enhanced cognitive performance. This finding suggests that BCI training, which has been shown to improve interhemispheric imbalance in the motor network of stroke patients [43], may also have the potential to enhance FPN function by modulating the balance of the bilateral PPCs.

However, we did not observe improvements in the efficiency of the alerting network. Based on previous studies [21], we suggest that this may be due to insufficient training intensity. Greater training volume might be needed to induce significant changes in brain function and thereby enhance behavioral performance.

### 4.3. BCI Training Induced Optimization of the Connectivity Within FPNs Related to Task Performance

The FPNs are the neural basis of alerting, orienting, and executive control functions. These three components are both relatively independent and interact with each other in terms of anatomy and functions [4]. In the resting state, our findings revealed that the EC from right PPC to left PPC was significantly decreased. An upward trend in EC from the right PPC to the left PFC was observed. In addition to the right PPC, the left PPC also contributes to visuospatial tasks to a certain extent. Studies have shown that complex visuospatial tasks can induce co‐activation of the bilateral PPCs [40]. In our study, the ANT is a relatively simple attention task that does not require substantial cognitive resources. BCI training reduced the EC from the right PPC to the left PPC but enhanced the EC from the right PPC to the left PFC, indicating that it promoted dynamic changes in task‐related brain networks [44], thereby leading to more efficient cognitive resource utilization.

We simultaneously used fNIRS to examine the connectivity of the FPNs during the ANT. Our findings revealed significant increases in EC from the right PFC to the right PPC, while EC from the left PFC to the left PPC was significantly reduced. These results suggest that BCI training induces task‐related optimization and reorganization of connectivity within FPNs [45, 46]. Prior research has demonstrated that the brain dynamically adjusts functional connectivity in response to task demands [39]. Specifically, the brain may reduce connectivity in certain regions while enhancing connectivity between key regions to improve overall efficiency. The FPNs exhibit the right‐hemispheric lateralization [47]. After BCI training, we observed a significant enhancement in EC within the right FPN, which may represent a potential neural mechanism underlying the improvement in FPN’s function. Consistent with previous studies on cortical localization during motor imagery, our results confirm the involvement of the sensorimotor cortex and PFC in motor imagery tasks. Enhanced plasticity in these regions is associated with improved cognitive control of BCI [18]. For instance, a functional MRI study reported that BCI training increases connectivity among the thalamus, PFC, and cerebellum. These regions, as integral components of the cerebellar‐thalamocortical neural pathway, are implicated in executive function [48]. Additionally, connectivity between the thalamus and the PFC has been shown to play a role in attention processing [49]. Therefore, we propose that the directed connectivity between the right PFC and right PPC represents a potential neural circuit underlying executive control. BCI training may enhance executive function by strengthening this connectivity.

### 4.4. Clinical Implications

The experimental device utilized in our study was a noninvasive BCI. Participants reported no discomfort or pain during or after the experimental procedures. For healthy young individuals, our findings provide a convenient and efficient cognitive training modality. Studies have shown that neural networks involved in attention and executive function overlap with brain regions associated with intelligence, highlighting the importance of attention networks for academic learning related to intellectual development [50]. For healthy older adults, BCI training may help mitigate age‐related cognitive decline and holds potential for the prevention of neurodegenerative diseases, such as Alzheimer’s disease.

In patients with ADHD, the disconnection within brain networks is a critical factor contributing to inattention and executive dysfunction [51]. Our study demonstrates that BCI training promotes the normalization of brain networks, thereby holding potential for improving ADHD symptoms. Specifically, BCI training optimizes connectivity patterns within the FPNs, revealing a distinct capacity to facilitate neuroplasticity. These findings provide a theoretical basis for the application of BCI training in the rehabilitation of attention and executive function deficits associated with neurologic disorders such as stroke and ADHD.

### 4.5. Limitations

Firstly, we did not include a sham stimulation condition in our study. Secondly, our participants were healthy young college students who had received higher education and possessed certain cognitive abilities. Future research should extend this study to a broader range of age groups and individuals with different diseases.

## 5. Conclusion

Our study provides evidence that a single session of BCI training can significantly enhance the efficiency of the executive control network in healthy young people. BCI training optimizes connectivity within the FPNs, which may be the mechanism of enhancing executive control function. These results suggest that BCI training may serve as an effective cognitive intervention for improving attention and executive function. Future research should investigate the long‐term effects of BCI training and explore its potential application in clinical populations, such as patients with ADHD and stroke.

## Conflicts of Interest

The authors declare no conflicts of interest.

## Author Contributions

**Yuhong Huang:** writing – review and editing, writing – original draft, visualization, software, methodology, investigation, formal analysis, data curation. **Qian Ding:** writing – review and editing, methodology, investigation, formal analysis, data curation. **Zhenghong Chen:** writing – review and editing, methodology, investigation, formal analysis, data curation. **Jing Chen:** methodology, investigation. **Yawen Li:** methodology, investigation. **Lu Chen:** methodology, investigation. **Shantong Yao:** methodology, investigation. **Yue Lan:** supervision, resources, project administration, conceptualization. **Guangqing Xu:** writing – review and editing, resources, project administration, funding acquisition, conceptualization. Yuhong Huang, Qian Ding, and Zhenghong Chen contributed equally to this work and share the first authorship.

## Funding

This work was supported by the National Natural Science Foundation of China (Grants 82272588 [Guangqing Xu], 82572917 [Guangqing Xu], 82072548 [Guangqing Xu], 82472619 [Yue Lan], and 82102678 [Qian Ding]), the Guangzhou Municipal Science and Technology Program (Grants 202206010197 [Yue Lan], 202201020378 [Yue Lan], and 2024A04J3082 [Qian Ding]), the Natural Key Research and Development Program of China (Grant 2022YFC2009700 [Yue Lan]), and the Guangdong Medical Research Foundation (Grant A2024500 [Qian Ding]).

## Data Availability

The data that support the findings of this study are available from the corresponding author upon reasonable request.
